# The Response of Airborne Mycobiome to Dust Storms in the Eastern Mediterranean

**DOI:** 10.3390/jof7100802

**Published:** 2021-09-25

**Authors:** Xuefeng Peng, Daniela Gat, Adina Paytan, Yinon Rudich

**Affiliations:** 1 School of Earth, Ocean and Environment, University of South Carolina, Columbia, SC 29208, USA; 2 Department of Earth and Planetary Sciences, Weizmann Institute of Science, Rehovot 76100, Israel; daniela.gat@weizmann.ac.il; 3 Joint Mass Spectrometry Centre (JMSC) of Comprehensive Molecular Analytics (CMA), Helmholtz Zentrum München–German Research Center for Environmental Health GmbH, 81379 Munich, Germany; 4 Institute of Marine Science, University of California, Santa Cruz, CA 95064, USA; apaytan@ucsc.edu

**Keywords:** airborne fungi, dust storms, fungal diversity, metagenome, metagenome-assembled genome, cladosporiales, aerobiome

## Abstract

Airborne microbial communities directly impact the health of humans, animals, plants, and receiving ecosystems. While airborne bacterial and fungal communities have been studied by both cultivation-based methods and metabarcoding surveys targeting specific molecular markers, fewer studies have used shotgun metagenomics to study the airborne mycobiome. We analyzed the diversity and relative abundance of fungi in nine airborne metagenomes collected on clear days (“background”) and during dust storms in the Eastern Mediterranean. The negative correlation between the relative abundance of fungal reads and the concentrations of atmospheric particulate matter having an aerodynamic diameter smaller than 10 μm (PM10) indicate that dust storms lower the proportion of fungi in the airborne microbiome, possibly due to the lower relative abundance of fungi in the dust storm source regions and/or more effective transport of bacteria by the dust. Airborne fungal community composition was altered by the dust storms, particularly those originated from Syria, which was enriched with xerophilic fungi. We reconstructed a high-quality fungal metagenome-assembled genome (MAG) from the order Cladosporiales, which include fungi known to adapt to environmental extremes commonly faced by airborne microbes. The negative correlation between the relative abundance of Cladosporiales MAG and PM10 concentrations indicate that its origin is dominated by local sources and likely includes the indoor environments found in the city.

## 1. Introduction

Bioaerosols, including cellular material and proteins, can constitute up to 25% of the atmospheric aerosol and, upon deposition, can affect many environmental processes in receiving ecosystems [1,2,3,4]. Bacteria, fungi, and viruses, including those pathogenic to humans, plants, and animals, can be transported over long distances [5,6,7,8]. Airborne microorganisms can act as ice nucleation surfaces for cloud formation and are predicted to play an increasingly important role compared to mineral particles due to global warming [9,10].

The diversity of airborne bacteria and fungi have been investigated by both cultivation-based methods and, more recently, metabarcoding surveys [11,12,13,14,15]. While these studies have advanced our understanding of microbial communities present in the atmosphere, it should be noted that both cultivation-based and metabarcoding surveys have inherent biases. Cultivation-based methods are biased against microorganisms recalcitrant to cultivation, and metabarcoding surveys are biased by the selection of primers for polymerase chain reactions (PCR). Shotgun metagenomics is a culture-independent method that does not suffer from the same type of PCR bias as metabarcoding surveys that target specific molecular markers such as part of the ribosomal RNA (both small and large units) genes and the internal transcribed spacers. Shotgun metagenomics has been broadly applied to study aquatic and terrestrial systems, but the number of airborne metagenomics studies remains small [16]. This is particularly true for the study of fungi in airborne communities because they typically are outnumbered by bacteria [17] and therefore require high sequencing depth during shotgun metagenome sequencing [18,19]. Recent advances in bioinformatics are enabling the reconstruction of eukaryotic genomes from complex microbial communities [20,21], but these methods have not been applied to airborne metagenomes.

Resulting from strong turbulent winds, dust storms can transport large quantities of particulate matter from desert surfaces over thousands of kilometers [22]. Dust storms frequently occur in the Eastern Mediterranean [23] and are known to transport high loadings of mineral particulate matter and airborne microbes and impact local bacterial communities [24,25,26]. Moreover, changes in the atmospheric microbiome linked with dust-storms from different sources, sampled at a single location, were observed by Gat et al. (2017), using 16S amplicon sequencing, yet no similar study was conducted for fungal taxa, to the best of our knowledge. In this study, we investigated the fungal diversity in airborne microbial communities in the Eastern Mediterranean and its response to dust storms. Fungal community composition on clear days (“background”) and during dust storms was determined by classifying and comparing shotgun metagenomic reads. Additionally, we reconstructed the first fungal metagenome-assembled genome from airborne metagenomes, which belongs to the recently proposed order Cladosporiales.

## 2. Materials and Methods

### 2.1. Sample Collection

Sample collection details were published in a recent study [24]. Briefly, particulate matter with an aerodynamic diameter smaller than 10 µm (PM10) was collected on pre-baked quartz fiber filters on the roof of a four-story building in Rehovot, Israel (31.9070N, 34.8102E, 80 m above sea level). The filters were stored frozen in sterile aluminum foil until nucleic acid isolation. The source of each sample was determined using the hybrid single-particle Lagrangian integrated trajectory model (HYSPLIT) [27] and verified using remote sensing images from the Giovanni online data system [28] (Supplementary Figure S1). The aerosol concentrations of atmospheric PM10 were retrieved from the Israeli Ministry of Environmental Protection database, Rehovot Air Monitoring station, situated ca. 1 km from the sampling station (https://www.svivaaqm.net/ (accessed on 24 September 2021)). All sampled events are listed in Supplementary Table S1.

### 2.2. DNA Extraction and Sequencing

The isolation of DNA from PM10 particles collected on filters has been described in detail [24]. In short, the DNeasy PowerSoil Kit (QIAGEN, Germany) with some modifications was used to extract DNA. Due to low DNA yield, samples from similar source aerosols and with similar PM10 concentrations were pooled (Supplementary Table S1), resulting in three samples representing two major dust events originated in Syria (“Syria_d1 (Dust_6)”, “Syria_d2 (Dust_7)”, and “Syria_d3 (Dust_8)”) and two samples representing aerosols from the Arabian Desert (“Arabia_d1 (Dust_9)” and “Arabia_d2 (Dust_10)”). Three composite samples representing moderate dust events, one from the Sahara (“Dust 3, North Africa”) and two of mixed dust sources (“Dust 4 and 5”), were also extracted. Additionally, a single composite sample (“Dust 2”) representing days with low PM10 concentrations (≤44 µg m^−3^, mean daily PM10 load in Israel [29]) was defined as background non-dust storm conditions, similarly to Gat et al. [24]. For negative control, a blank filter was processed along with all other samples. DNA quantity and quality was assessed using High Sensitivity D1000 ScreenTape (Agilent Technologies, Santa Clara, CA, USA); DNA quantities are provided in Table S1. Blank control showed no presence of DNA and therefore was not further processed. Libraries for shotgun metagenome sequencing were prepared with the Accel-NGS 1S Kit (Swift Biosciences, Ann Arbor, MI, USA) and size selected (375–425 bp) using Pippin-prep (Sage Science, Beverly, MA, USA). A single lane on a HiSeq2500 platform (Illumina, San Diego, CA, USA) was used to sequence the nine samples with 2 × 250 cycles.

### 2.3. Analysis of Metagenomes

Raw reads were first filtered using the tool BBDuk Version 38.73 [30] with the options “ktrim = r ordered minlen = 101 minlenfraction = 0.33 mink = 11 tbo tpe rcomp = f k = 23 ftm = 5”. Adapters were trimmed from the BBDuk-filtered reads using the tool Trimmomatic Version 0.39 [31] with the options “ILLUMINACLIP:$adapters:2:30:10 LEADING:3 TRAILING:3 SLIDINGWINDOW:4:15 MINLEN:150”. Paired reads after quality filtering and adapter trimming were merged using the tool BBMerge Version 38.73 [30] with default options. Merged reads were queried against the NCBI nr database using DIAMOND [32] with an e-value threshold of 1 × 10^−5^. The resultant NCBI taxonomy ID was used to assign taxonomy to each read. Given the rapidly evolving nature of fungal taxonomy in the past decade [33,34], there is not a single “standard” classification scheme for the fungal kingdom. Here we adopt the phylogenomics-based classification from the Fungal Tree of Life [33]. The normalized fungal abundance for a given fungal taxon (f) was calculated as:

$$f=\frac{N}{D\times G}$$

where N is the number of reads classified to this fungal taxon, D is the total number of reads from this sample (sequencing “depth”), and G is the average genome size of this fungal taxon determined from Mycocosm [35]. The relative abundance of a given fungal taxon in a sample (r) was calculated as:

$$r=\frac{f}{\sum f}$$

where f is the normalized fungal abundance of this fungal taxon, and ∑f is the sum of the normalized fungal abundance of all fungal taxa in this sample.

### 2.4. Fungal Metagenome-Assembled Genome

Co-assembly of all nine metagenomes was performed with MEGAHIT (v1.2.9) [36] using kmers 127, 137, 147, 157, 167, 177, 187, 197, 207, 217, and 227. Putative eukaryotic contigs greater than 1500 bp in size were selected by the software EukRep [20]. Unsupervised binning was performed with CONCOCT [37] and Metabat2 [38], which were implemented by metaWRAP [39]. This resulted in five eukaryotic bins greater than 10 Mbp in size. The completeness of these putative eukaryotic metagenome-assembled genomes (MAGs) was estimated by benchmarking the number of universal single-copy orthologs to those common to the fungal kingdom using the tool BUSCO v5.2.1 [40,41] archaeal, bacterial, and viral orthologs with the option “--auto-lineage-euk”. One of the five MAGs was greater than 50% in completeness and was used for downstream analysis. BUSCO determined that the MAG belongs to the order Capnodiales under the fungal kingdom. Quality-filtered reads from each metagenome library were mapped to the MAG using BBMap [30] to determine its coverage in each metagenome.

The taxonomy of the fungal MAG was determined by placing it in a phylogenomic tree including 45 Ascomycota genomes as references, most of which from the class Dothideomycetes. All reference genomes were downloaded from NCBI GenBank or the Joint Genome Institute Genome Portal. Single-copy orthologs common to the phylum Ascomycota were identified with the tool BUSCO v5.2.1 [40,41] archaeal, bacterial, and viral orthologs. A total of 74 single-copy orthologs, which were found in at least 44 of the 46 genomes analyzed, were aligned by MUSCLE v3.8.31 [42], concatenated, and trimmed by the tool trimAl v1.2 [43] with the option “-gt 0.9 -cons 60 -w 3”. Trimmed alignment of the concatenated protein sequences were used to construct a phylogenomic tree with FastTree v2.1.11 [44]. Visualization of the tree was performed in MEGA X [45].

This fungal MAG was submitted to NCBI GenBank for contamination screening. A total of 26 contigs identified by the NCBI as contaminants were removed. Gene prediction and annotation was performed using the funannotate pipeline [46]. Specifically, contigs were masked with the method TANTAN [47]. Structural annotation was performed by Augustus [48], SNAP [49], and GlimmerHMM [50]. The best gene models were selected by EVidenceModeler [51]. tRNAs were predicted with tRNAscan [52]. Functional annotations of predicted genes were performed with Interproscan v5.51−85.0 [53] and eggNOG-mapper [54,55]. CAZymes were predicted with dbCAN2 [56], and signal peptides were predicted with SinalP 5.0 [57]. The fungal MAG can be accessed by NCBI BioProject number PRJNA746316.

### 2.5. Statistical Analysis

To investigate the relationship between PM10 concentration and the relative abundance of fungal reads, Spearman’s rank-order correlation coefficient (rho) and the associated *p*-value was calculated in R [58]. Non-metric multidimensional scaling (NMDS) was performed using the fungal relative abundance data in R and visualized with the ggplot2 package [59]. To test the null hypothesis that the fungal community composition was not affected by the origin of dust events, we performed analysis of similarities (ANOSIM) with 9999 free permutations.

## 3. Results

**Figure 1:**
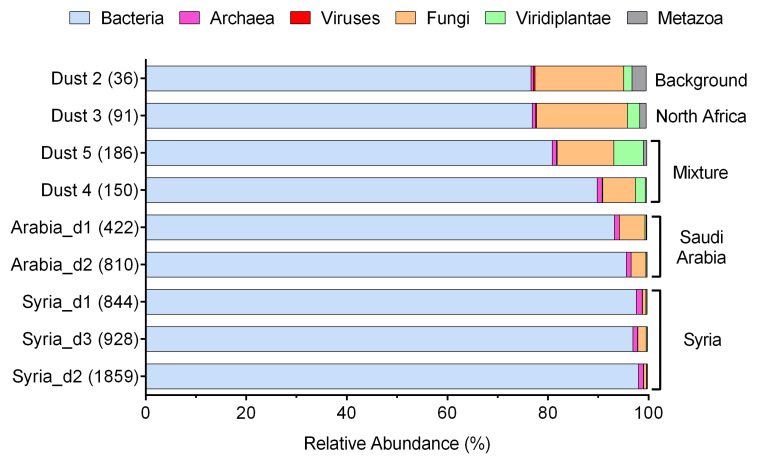
The relative abundance of reads classified by DIAMOND as bacteria, archaea, viruses, fungi, Viridiplantae, and Metazoa in aerosol samples. The numbers in the parenthesis behind the sample number represent PM10 concentrations in μg m^−3^. The origin of the samples is marked to the right of the stacked bars. Sample “Dust 4” includes a mixture of aerosols originated from North Africa and Saudi Arabia. Sample “Dust 5” includes a mixture of aerosols originated from North Africa, Jordan, and Sinai Peninsula.

**Figure 2:**
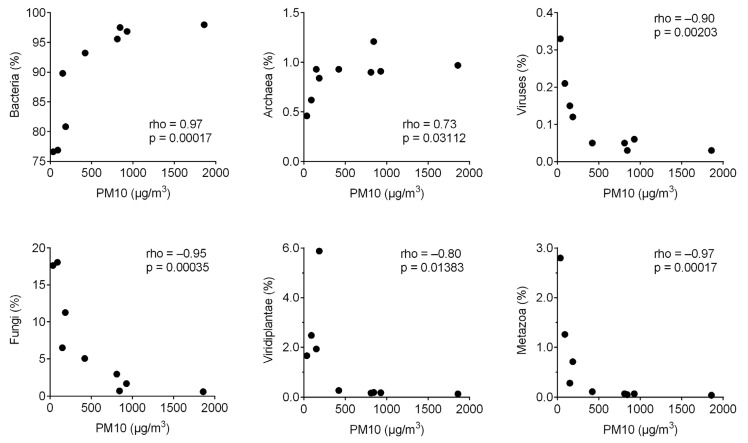
The relationships between the concentration of atmospheric particulate matter having an aerodynamic diameter smaller than 10 μm (PM10) and the relative abundance of reads classified as Bacteria, Archaea, Viruses, Fungi, Viridiplantae, and Metazoa. Spearman’s rank-order correlation coefficient (rho) and the associated *p*-value (p) are reported in each panel.

Of the nine metagenomes, approximately half (41–54% with an average of 48%) of the reads that passed quality filtering returned a positive hit from DIAMOND queries (Supplementary Figure S2). Bacteria dominated the reads classified by DIAMOND, while fungi accounted for 0.6–18% of the classified reads (Figure 1). The relative abundance of Viridiplantae was extremely low (<0.3%) in dust storm samples that originated from Syria and Saudi Arabia but ranged from 1.7–5.9% in the other samples. The relative abundance of Bacteria and Archaea was positively correlated with PM10 concentrations, whereas the relative abundance of fungi, viruses, Viridiplantae, and Metazoa was negatively correlated with PM10 concentrations (Figure 2).

**Figure 3:**
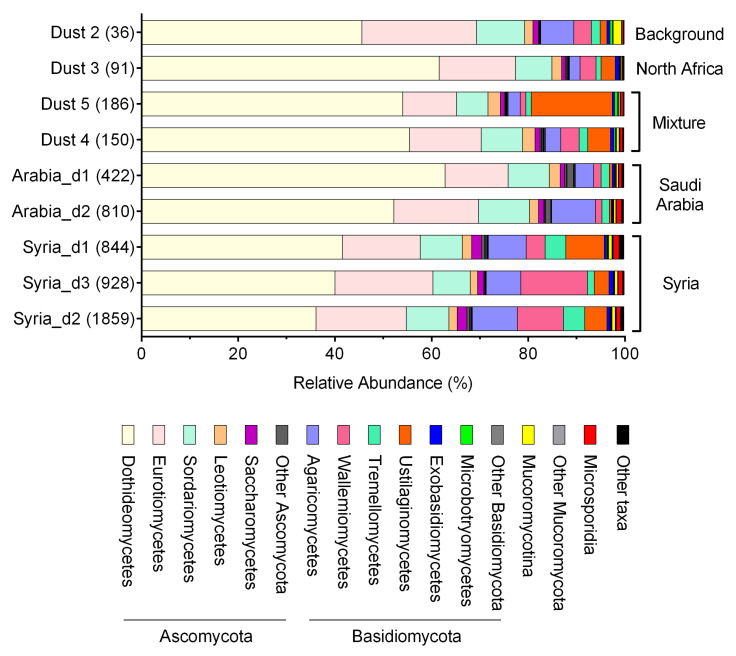
The relative abundance of fungal reads classified by DIAMOND at the class level (except for Mucoromycotina and Microsporidia) in aerosol samples. The numbers in the parenthesis behind the sample number represent PM10 concentrations in μg m^−3^. The origin of the samples is marked to the right of the stacked bars. Sample “Dust 4” includes a mixture of aerosols originated from North Africa and Saudi Arabia. Sample “Dust 5” includes a mixture of aerosols originated from North Africa, Jordan, and Sinai Peninsula.

**Figure 4:**
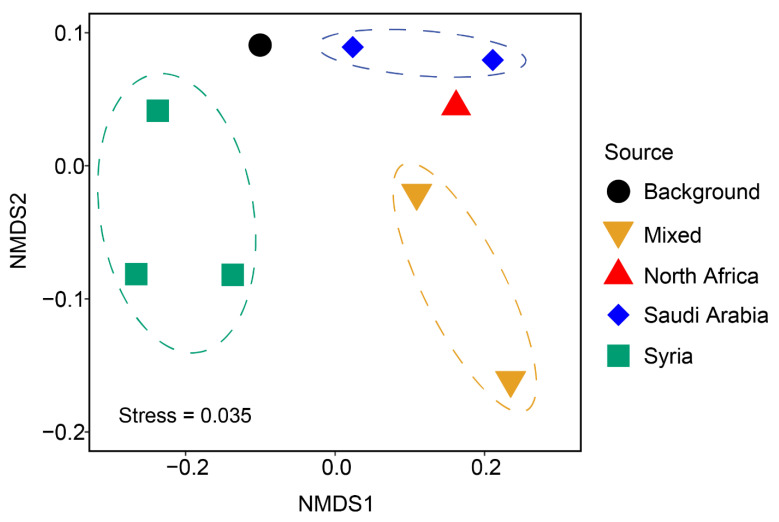
Non-metric multidimensional scaling (NMDS) ordination plot based on the fungal diversity at the class level. The Bray–Curtis distance was calculated and used for the NMDS analysis. The black circle represents samples collected locally (“Background”). The red triangle represents a sample originated from North Africa. Blue diamonds represent samples originated from Saudi Arabia. Green squares represent samples originated from Syria. Yellow inverted triangles represent “Mixed” samples originated from North Africa and other locations (see Supplementary Table S1 for details). The test statistics (R) from the analysis of similarities (ANOSIM) with 9999 free permutations was 0.7537, with a *p*-value of 0.0025.

Ascomycota was the dominant fungal phylum in all samples, accounting for 68–90% of fungal reads (Figure 3 and Supplementary Table S2). Dothideomycetes was the most abundant Ascomycota class, followed by Eurotiomycetes, Sordariomycetes, Leotiomycetes, and Saccharomycetes (paired *t*-test two-tailed *p*-value < 0.05). The relative abundance of the second most abundant fungal phylum, Basidiomycota, was lower in the dust samples from the Sahara (“Dust 3, North Africa”) and of mixed dust sources (“Dust 4 and 5”) than in samples originated from Syria (Figure 3, *t*-test two-tailed *p*-value < 0.05). Among the basidiomycetous classes, the relative abundance of Ustilaginomycetes was the highest in the mixed sample originated from North Africa, Sinai, and Jordan (“Dust 5”). The relative abundance of Wallemiomycetes was particularly high in two samples from Syria (“Dust 7 and 8”) compared to the other samples (Supplementary Table S2). Across all samples, Agaricomycetes, Wallemiomycetes, Tremellomycetes, and Ustilaginomycetes were the abundant Basidiomycota classes. Many of the fungal classes are known spore-forming taxa. Early-diverging fungi accounted for a small fraction of the total fungal community, and they primarily consisted of Mucoromycotina and Microsporidia. Of the twenty most abundant fungal taxa, there was a positive correlation between Saccharomycetes, Agaricomycetes, Microsporidia, and Chytridiomycota and the PM10 concentrations (Table 1). The NMDS ordination plot showed that fungal community composition was differentiated by the origin of the aerosol samples (Figure 4), and the difference was statistically significant (ANOSIM *p*-value = 0.035).

**Figure 5:**
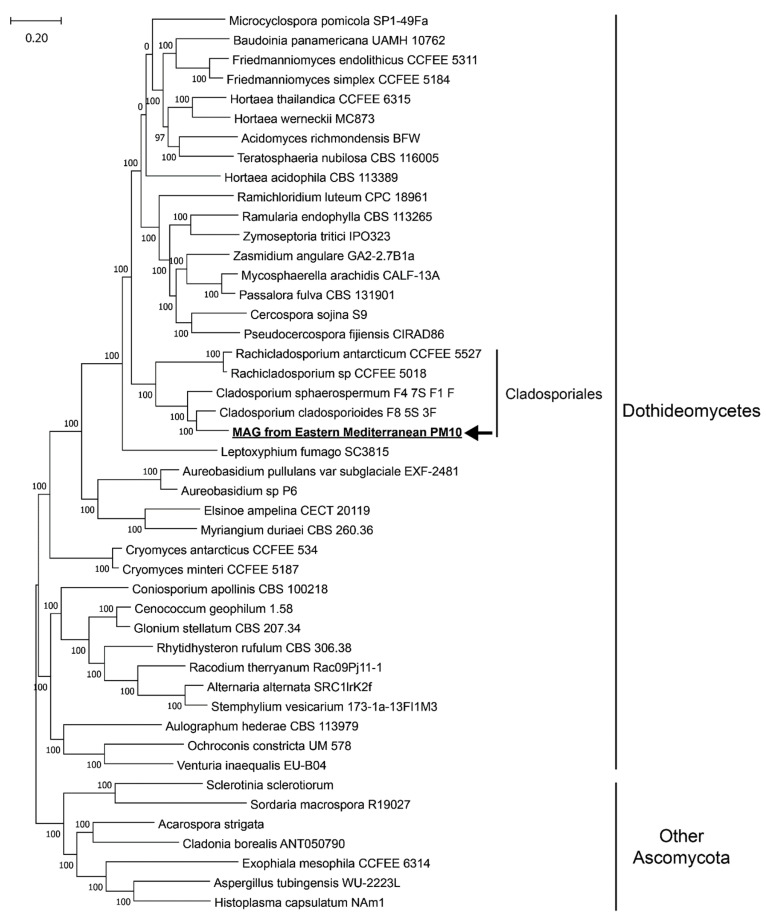
Phylogenomics tree including the fungal MAG reconstructed in this study (underlined and in bold and pointed out by an arrow) and 45 reference fungal genomes. Bootstrap values are shown at branching points of the tree.

The fungal MAG reconstructed from the nine metagenomes was 71.5% complete (38.8% complete and single-copy BUSCOs, 32.7% complete and duplicated BUSCOs, 5.0% fragmented BUSCOs, and 23.5% missing BUSCOs) as evaluated by the “capnodiales_odb10” dataset, which includes 3578 single-copy genes [60] protist, bacterial, and viral genomes for evolutionary and functional annotations of orthologs. The size of the fungal MAG is 49.78 Mbp with 13,574 contigs, and the GC content is 53.34%. The maximum contig length is 30.334 Kbp, and the N50 value is 3827 bp. The phylogenomic tree we constructed indicates that the fungal MAG should belong to the recently proposed order Cladosporiales (Figure 5) [61].

**Figure 6:**
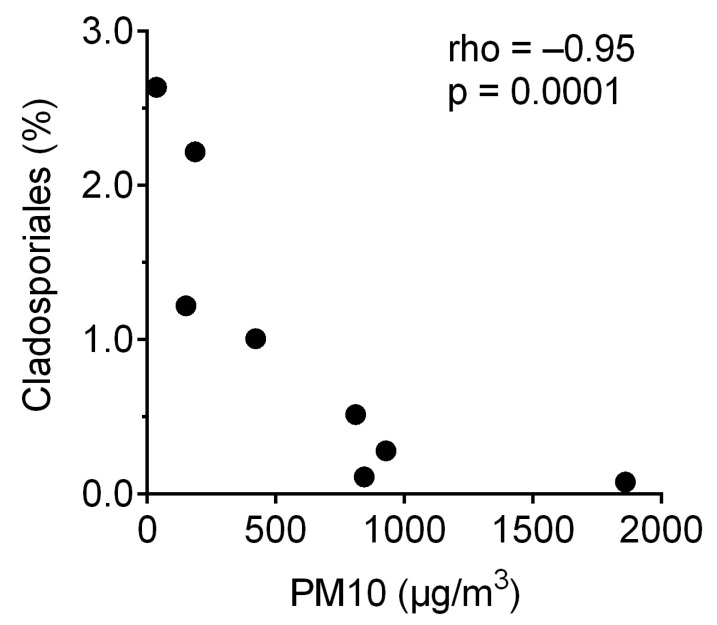
Percentage of reads from each metagenome that mapped to the fungal MAG reconstructed in this study.

The Cladosporiales MAG accounted for 0.1 to 2.6% of total reads in the metagenomic libraries, and the percentage of reads that mapped to the Cladosporiales MAG correlated inversely with PM10 concentrations (Figure 6). The highest Cladosporiales MAG abundance was found in the local background sample.

## 4. Discussion

Our data indicate that fungi can constitute up to 18% of the microbial community in the PM10 part of the air microbiome. This ratio is one to two orders of magnitude higher than what has been reported for the water column of the open ocean, where fungal reads were up to 0.2% of the total metagenomes [62]. This difference suggests that atmospheric transport is a potential mechanism for fungal dispersal from land to sea, an idea that has been suggested for bacterial dispersal [8,24], and is consistent with the positive correlation between the relative abundance of bacterial/archaeal reads and PM10 concentrations in this study (Figure 2). However, the negative correlation between the relative abundance of fungal reads and PM10 concentrations we observe points to a “dilution” effect of dust storms on the local airborne fungal community. The low relative abundance of fungal reads during dust storms is potentially a result of low fungal abundance at the origin locations of dust storms, assuming that prokaryotic cells and fungal spores are equally likely to be transported. Similar negative correlations between the relative abundance of metagenomic reads and PM10 concentrations were also observed for Viridiplantae and Metazoa, both of which are expected to have a lower relative abundance in desert soils from where the dust storms originated than in the local urban setting (Figure 2). Despite the classification of approximately half of the reads that passed quality filter (Supplementary Figure S2), we would like to point out that read-based metagenomic analysis is intrinsically limited by the quality of the database used (in our case, the NCBI nr database). Additionally, the metagenomic-based approach cannot determine the absolute abundance of microorganisms, which can be estimated by traditional cultivation techniques [63]. Future studies can incorporate methods that quantify the absolute abundance of the air microbiome such as cell count analysis [64].

Non-metric multidimensional scaling (NMDS) analysis revealed that dust storms not only reduced the relative abundance of fungi in the airborne microbial community but also shifted fungal community composition (Figure 4). For example, dust storms originated from Syria were enriched with Ustilaginomycetes (Figure 3), which include many plant pathogens [65]. A similar observation for bacterial taxa was shown by Gat et al. (2017), analyzing the bacterial taxonomy of the same two storms and showing that they differed in their bacterial community composition. In bacteria, it was observed that dust storms increased the relative abundance of soil-associated bacteria, compared with low PM10 conditions, which were characterized by anthropogenic-associated bacteria. However, the change in fungal taxa suggests that one potential detrimental effect of dust storms, in addition to public health concerns, is the elevated chance of inducing plant disease through transported fungi. Dust storms originated from Syria resulted in the highest PM10 concentrations and were particularly enriched with Wallemiomycetes, which includes many known xerophilic fungi [66]. The ability of Wallemiomycetes to tolerate low water conditions likely contributed to their high relative abundance in these strong dust storm events. However, dust storms that originated from Saudi Arabia carry similar fungal communities as the local background airborne fungal communities and hence are less likely to alter the local airborne fungal community. Although there was considerable variability in PM10 concentrations between the different days of the same dust storm originated in Syria and Saudi Arabia (Supplementary Table S1), the fungal community sampled from each day was relatively similar to each other (Figure 3 and Figure 4). This suggests the ability of dust storms to homogenize the aerial microbial community.

The reconstruction of a high-quality fungal metagenome-assembled genome (MAG) was enabled by the depth of our metagenome sequencing, the relatively high percentage of fungal reads, and the relatively high abundance of one lineage of fungi, which in this case was Cladosporiales (Dothideomycetes). Cladosporiales was recently proposed to be elevated from the Capnodiales family Cladosporiaceae based on the phylogenetic study of four nuclear loci [61]: partial 28S large subunit RNA gene (LSU), internal transcribed spacers and intervening 5.8S nuclear ribosomal DNA (ITS), partial translation elongation factor 1-alpha gene (TEF-1α), and partial RNA polymerase II second largest subunit gene (RPB2). Phylogenomics analysis showed that the fungal MAG reconstructed in this study likely belongs to the genus Cladosporium (Figure 5). Cladosporium is a common and diverse group of hyphomycete ubiquitous in the environment [13,67,68]. They adapt to extreme temperature and moisture conditions by developing conidia capable of rehydration and germination within hours [61]. Hardiness appears to be a typical trait within Cladosporiales, as another Cladosporiales genus with sequenced genomes, Rachicladosporium, are endolithic fungi found in Antarctica [69]. Therefore, the Cladosporiales MAG from this study is likely adapted to the environmental extremes associated with airborne microbial communities. The negative correlation between the percentage of reads mapping to the Cladosporiales MAG and PM10 concentrations (Figure 6) suggests a local origin of the fungi represented by the MAG. Since Cladosporium species are one of the most prevalent lineages in indoor environments [70], indoor environments local to the city of Rehovot may be a major source of airborne fungi, as well as other airborne microbes. It is also possible that the negative correlation between the percentage of reads mapping to the Cladosporiales MAG and PM10 concentrations simply reflects the overall negative correlation we observed between the relative abundance of fungal reads and PM10 concentrations.

## 5. Conclusions

We analyzed the diversity and relative abundance of fungi in nine airborne metagenomes collected on clear days (“background”) and during dust storms. The negative correlation between the relative abundance of fungal reads and PM10 concentrations indicate that dust storms lower the proportion of fungi in the airborne microbiome, probably due to the lower relative abundance of fungi in the dust storm sources and extensive transport of bacteria by the dust. Airborne fungal community composition was altered by dust storms, particularly those originated from Syria, which was enriched with xerophilic fungi. We reconstructed a high-quality fungal metagenome-assembled genome (MAG) from the order Cladosporiales, which include fungi known to adapt to environmental extremes commonly faced by airborne microbes. The negative correlation between the relative abundance of Cladosporiales MAG and PM10 concentrations indicate its origin, which likely includes the indoor environments found in the city.

## Figures and Tables

**Table 1:** The Spearman’s rank-order correlation coefficient (rho) between PM10 and the relative abundance of the 20 most abundant fungal classes (shown as average%). *p*-values smaller than 0.05 are highlighted with bold fonts.

Class	Average%	Rho	*p*-Value
Dothideomycetes	49.91%	−0.65	0.06656
Eurotiomycetes	16.78%	0.216667	0.5809
Sordariomycetes	8.55%	0.15	0.7081
Leotiomycetes	2.01%	−0.36667	0.3363
Saccharomycetes	1.23%	0.733333	**0.03112**
Pezizomycetes	0.40%	0.383333	0.3125
Lecanoromycetes	0.31%	0.333333	0.3853
Orbiliomycetes	0.15%	0.233333	0.5517
Taphrinomycetes	0.11%	−0.11667	0.7756
Xylonomycetes	0.10%	−0.11667	0.7756
Agaricomycetes	5.78%	0.75	**0.02549**
Ustilaginomycetes	4.72%	0.15	0.7081
Wallemiomycetes	4.66%	0.5	0.1777
Tremellomycetes	2.12%	0.333333	0.3853
Exobasidiomycetes	0.56%	−0.23333	0.5517
Microbotryomycetes	0.25%	−0.61667	0.08573
Pucciniomycetes	0.14%	0.183333	0.6436
Microsporidia	0.69%	0.833333	**0.008267**
Mucoromycotina	0.62%	0.216667	0.5809
Chytridiomycota	0.18%	0.766667	**0.02139**

## Data Availability

Raw reads and the fungal metagenome-assembled genome generated in this study are available at NCBI under BioProject PRJNA746316.

## Supplementary Materials

The following are available online at https://www.mdpi.com/article/10.3390/jof7100802/s1, Figure S1. The back trajectories of each storm sample as obtained from the hybrid single-particle Lagrangian integrated trajectory model. Each file is named by the date of the back trajectory, which corresponds to the dates listed in Table S1. Figure S2: The percentage of raw reads that did not pass quality filter (grey), passed quality filter but returned no result from DIAMOND queries against the NCBI nr database (light blue), and passed quality filter and returned results from DIAMOND queries (green). Table S1: Sample description of dust collected in Israel. Samples Dust_2 to Dust_5 were created by pooling DNA from several sampling events. Table S2: The relative abundance of fungal taxa in each sample.
