# Supplementary material for: The Response of Airborne Mycobiome to Dust Storms in the Eastern Mediterranean
Source: J Fungi (Basel). 2021 Sep 25;7(10):802. doi: 10.3390/jof7100802 (PMC8540267; doi:10.3390/jof7100802)
Supplement: Supplementary file 1 [file jof-07-00802-s001.zip › Air_Fungi_XP_v8_SI.pdf]

Supplementary Information for

The response of airborne mycobiome to dust storms in the Eastern Mediterranean

Xuefeng Peng [1,*], Daniella Gat [2], Adina Paytan [3], and Yinon Rudich [2,*]

[1]School of Earth, Ocean and Environment, University of South Carolina, Columbia, SC, USA
[2]Department of Earth and Planetary Sciences, Weizmann Institute of Science, Rehovot, Israel
[3]Institute of Marine Science, University of California, Santa Cruz, CA, USA

*Correspondence: xpeng@seoe.sc.edu; yinon.rudich@weizmann.ac.il

This file includes:

Supplementary Tables

Supplementary Figures

Supplementary Table S1. Sample description of dust collected in Israel. Samples Dust_2 to Dust_5 were created by pooling DNA from several sampling events.

Sample	DNA concentration (pg μl^{-1})	Aerosol origin	Sampling date	PM_{10} ($\mu g\ m^{-3}$)	Total air volume sampled (m^3)	Sampling Duration (Hours)
Dust_2	3390	Local	7/18/2016	33	3254	48
		North Africa	6/6/2016	35	3254	48
		North Africa	8/15/2016	35	1627	24
		Mix	8/31/2016	37	3254	48
		Arabia	8/26/2016	40	2983	44
Dust_3	1390	North Africa	1/17/2017	75	3254	48
		North Africa	5/9/2017	84	1627	24
		North Africa	1/7/2017	96	3254	48
		North Africa	11/20/2016	109	1627	24
Dust_4	5770	Mix (North Africa & Arabia)	11/25/2016	121	1627	24
		North Africa	1/9/2017	144	3254	48
		North Africa	3/12/2017	150	1898	28
		Mix (North Africa & Arabia)	11/24/2016	186	3254	48
Dust_5	3270	North Africa	2/28/2016	209	2034	30
		Mix (Sinai Peninsula & Jordan)	11/23/2016	232	1424	21
		North Africa	4/12/2017	118	3254	48
Syria_d1 (Dust_6)	7130	Syria	9/8/2015	844	949	14
Syria_d2 (Dust_7)	10200	Syria	9/9/2015	1859	983	14.5
Syria_d3 (Dust_8)	3490	Syria	9/10/2015	928	1305	19.25
Arabia_d1 (Dust_9)	10500	Arabia	11/3/2015	422	1627	24
Arabia_d2 (Dust_10)	10200	Arabia	11/4/2015	810	1627	24

Supplementary Table S2. The relative abundance of fungal taxa in each sample.

	Dust_2	Dust_3	Arabia_d2 (Dust_10)	Arabia_d1 (Dust_9)	Syria_d3 (Dust_8)	Syria_d2 (Dust_7)	Syria_d1 (Dust_6)	Dust_5	Dust_4
**Source**	Background	North Africa	Saudi Arabia	Saudi Arabia	Syria	Syria	Syria	Mixed	Mixed
**Ascomycota**	**82.6%**	**88.5%**	**84.8%**	**89.7%**	**71.3%**	**68.4%**	**71.7%**	**75.8%**	**83.5%**
Dothideomycetes	45.6%	61.5%	52.2%	62.8%	40.0%	36.1%	41.6%	54.0%	55.4%
Eurotiomycetes	23.7%	15.8%	17.5%	13.0%	20.3%	18.7%	16.0%	11.1%	14.8%
Sordariomycetes	10.0%	7.5%	10.5%	8.5%	7.7%	8.8%	8.7%	6.5%	8.6%
Leotiomycetes	1.7%	2.0%	1.9%	2.3%	1.5%	1.7%	2.0%	2.6%	2.5%
Saccharomycetes	1.1%	0.7%	1.1%	0.9%	1.3%	2.0%	2.0%	0.8%	1.0%
Lecanoromycetes	0.2%	0.3%	0.2%	0.3%	0.2%	0.5%	0.6%	0.2%	0.3%
Orbiliomycetes	0.1%	0.1%	0.2%	0.2%	0.1%	0.2%	0.2%	0.1%	0.2%
Pezizomycetes	0.1%	0.2%	1.0%	1.3%	0.1%	0.2%	0.3%	0.1%	0.2%
Xylonomycetes	0.1%	0.1%	0.1%	0.1%	0.1%	0.1%	0.1%	0.1%	0.1%
Arthoniomycetes	0.0%	0.0%	0.0%	0.0%	0.0%	0.0%	0.0%	0.0%	0.1%
Neolectomycetes	0.0%	0.0%	0.0%	0.0%	0.0%	0.0%	0.0%	0.0%	0.0%
Pneumocystidomycetes	0.0%	0.0%	0.0%	0.0%	0.0%	0.0%	0.0%	0.0%	0.0%
Schizosaccharomycetes	0.0%	0.0%	0.0%	0.0%	0.0%	0.0%	0.0%	0.0%	0.0%
Taphrinomycetes	0.0%	0.1%	0.1%	0.2%	0.0%	0.1%	0.1%	0.1%	0.2%
**Basidiomycota**	**15.0%**	**10.6%**	**12.9%**	**8.5%**	**26.6%**	**28.9%**	**24.9%**	**22.9%**	**14.8%**
Agaricomycetes	6.8%	2.2%	9.1%	3.8%	7.2%	9.3%	7.8%	2.5%	3.2%
Wallemiomycetes	3.6%	3.3%	1.3%	1.5%	13.7%	9.5%	3.9%	1.2%	3.8%
Tremellomycetes	1.9%	1.0%	1.6%	1.7%	1.4%	4.4%	4.3%	1.1%	1.8%

Ustilaginomycetes	1.4%	2.9%	0.4%	0.6%	3.1%	4.6%	8.0%	16.7%	4.7%
Exobasidiomycetes	0.6%	0.8%	0.2%	0.5%	0.9%	0.6%	0.4%	0.5%	0.7%
Microbotryomycetes	0.5%	0.2%	0.1%	0.1%	0.1%	0.1%	0.2%	0.6%	0.4%
Agaricostilbomycetes	0.00004%	0.0%	0.0%	0.0%	0.0%	0.0%	0.0%	0.0%	0.0%
Bartheletiomycetes	0.0%	0.0%	0.0%	0.0%	0.0%	0.0%	0.0%	0.0%	0.0%
Cystobasidiomycetes	0.0%	0.0%	0.0%	0.0%	0.0%	0.0%	0.0%	0.0%	0.0%
Dacrymycetes	0.0%	0.0%	0.0%	0.0%	0.0%	0.1%	0.1%	0.0%	0.0%
Malasseziomycetes	0.0%	0.0%	0.0%	0.0%	0.0%	0.1%	0.1%	0.0%	0.0%
Mixiomycetes	0.0%	0.0%	0.0%	0.0%	0.0%	0.0%	0.0%	0.0%	0.0%
Pucciniomycetes	0.1%	0.1%	0.1%	0.2%	0.1%	0.1%	0.1%	0.2%	0.2%
**Mucoromycota**	**1.8%**	**0.4%**	**0.6%**	**0.5%**	**0.7%**	**0.9%**	**1.1%**	**0.4%**	**0.7%**
Mucoromycotina	1.7%	0.3%	0.5%	0.4%	0.6%	0.6%	0.7%	0.3%	0.5%
Endogonomycetes	0.0%	0.0%	0.0%	0.0%	0.0%	0.0%	0.1%	0.0%	0.0%
Glomeromycotina	0.0%	0.0%	0.1%	0.0%	0.0%	0.1%	0.1%	0.1%	0.1%
Mortierellomycotina	0.0%	0.0%	0.1%	0.1%	0.0%	0.1%	0.2%	0.1%	0.1%
**Zoopagomycota**	**0.1%**	**0.1%**	**0.1%**	**0.1%**	**0.1%**	**0.3%**	**0.3%**	**0.1%**	**0.1%**
Entomophthoromycotina	0.0%	0.0%	0.0%	0.1%	0.0%	0.1%	0.1%	0.0%	0.0%
Kickxellomycotina	0.0%	0.0%	0.0%	0.0%	0.0%	0.1%	0.1%	0.0%	0.0%
Zoopagomycotina	0.0%	0.0%	0.1%	0.1%	0.1%	0.1%	0.1%	0.0%	0.1%
**Chytridiomycota**	**0.1%**	**0.1%**	**0.2%**	**0.2%**	**0.1%**	**0.3%**	**0.5%**	**0.1%**	**0.1%**
Chytridiomycetes	0.1%	0.1%	0.2%	0.2%	0.1%	0.3%	0.5%	0.1%	0.1%
Monoblepharidomycetes	0.0%	0.0%	0.0%	0.0%	0.0%	0.0%	0.0%	0.0%	0.0%
Neocallimastigomycetes	0.0%	0.0%	0.0%	0.0%	0.0%	0.0%	0.0%	0.0%	0.0%
**Cryptomycota**	**0.0%**	**0.0%**	**0.0%**	**0.0%**	**0.0%**	**0.1%**	**0.1%**	**0.0%**	**0.0%**

**Blastocladiomycota**	**0.0%**	**0.0%**	**0.1%**	**0.0%**	**0.0%**	**0.0%**	**0.0%**	**0.0%**	**0.0%**
**Microsporidia**	**0.3%**	**0.2%**	**1.0%**	**0.6%**	**0.9%**	**0.9%**	**1.1%**	**0.5%**	**0.5%**
Others	0.2%	0.2%	0.3%	0.2%	0.2%	0.2%	0.3%	0.2%	0.2%

Supplementary Figure S1. (Separate pdf files) The back trajectories of each storm sample as obtained from the hybrid single-particle Lagrangian integrated trajectory model (Stein, A. F.; Draxler, R. R.; Rolph, G. D.; Stunder, B. J. B.; Cohen, M. D.; Ngan, F. NOAA's HYSPLIT Atmospheric Transport and Dispersion Modeling System. Bull. Am. Meteorol. Soc. 2015, 96, 2059−2077). Each file is named by the date of the back trajectory, which corresponds to the dates listed in Supplementary Table S1.

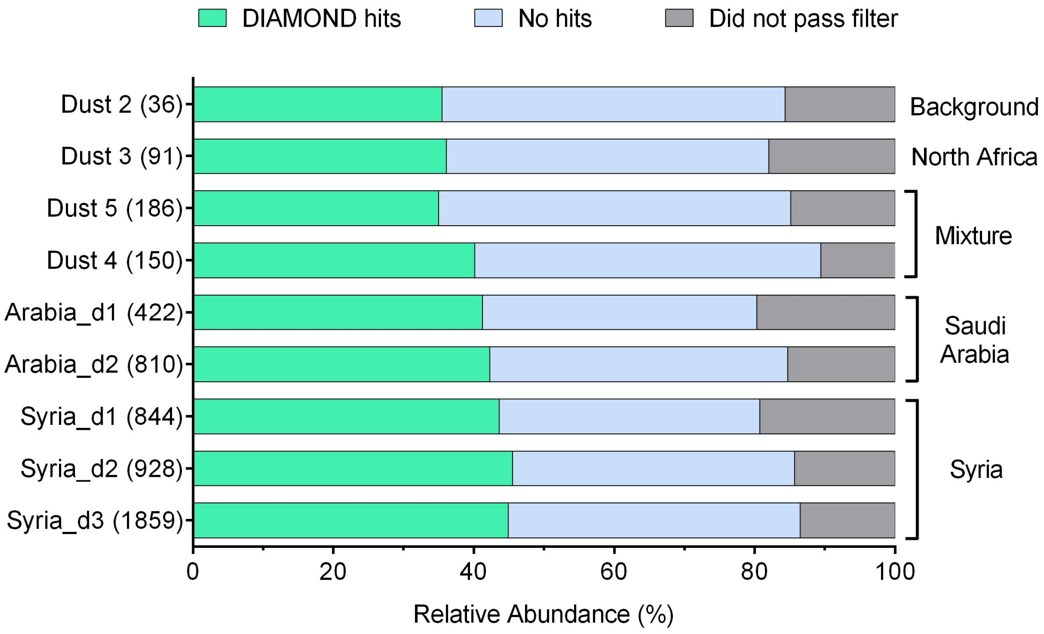

Supplementary Figure S2. The percentage of raw reads that did not pass quality filter (grey), passed quality filter but returned no result from DIAMOND queries against the NCBI nr database (light blue), and passed quality filter and returned results from DIAMOND queries (green). The numbers in the parenthesis behind the sample number represent PM10 concentrations in μg m^{-3}. The origin of the samples is marked to the right of the stacked bars. Sample "Dust 4" includes a mixture of aerosols originated from North Africa and Saudi Arabia. Sample "Dust 5" includes a mixture of aerosols originated from North Africa, Jordan, and Sinai Peninsula.
