# Supplementary material for: The Response of Airborne Mycobiome to Dust Storms in the Eastern Mediterranean
Source: J Fungi (Basel). 2021 Sep 25;7(10):802. doi: 10.3390/jof7100802 (PMC8540267; doi:10.3390/jof7100802)
Supplement: Supplementary file 1 [file jof-07-00802-s001.zip › Supplementary_Fig_S1.zip!/2017-01-09.pdf]

NOAA HYSPLIT MODEL

Backward trajectories ending at 0600 UTC 09 Jan 17

GDAS Meteorological Data

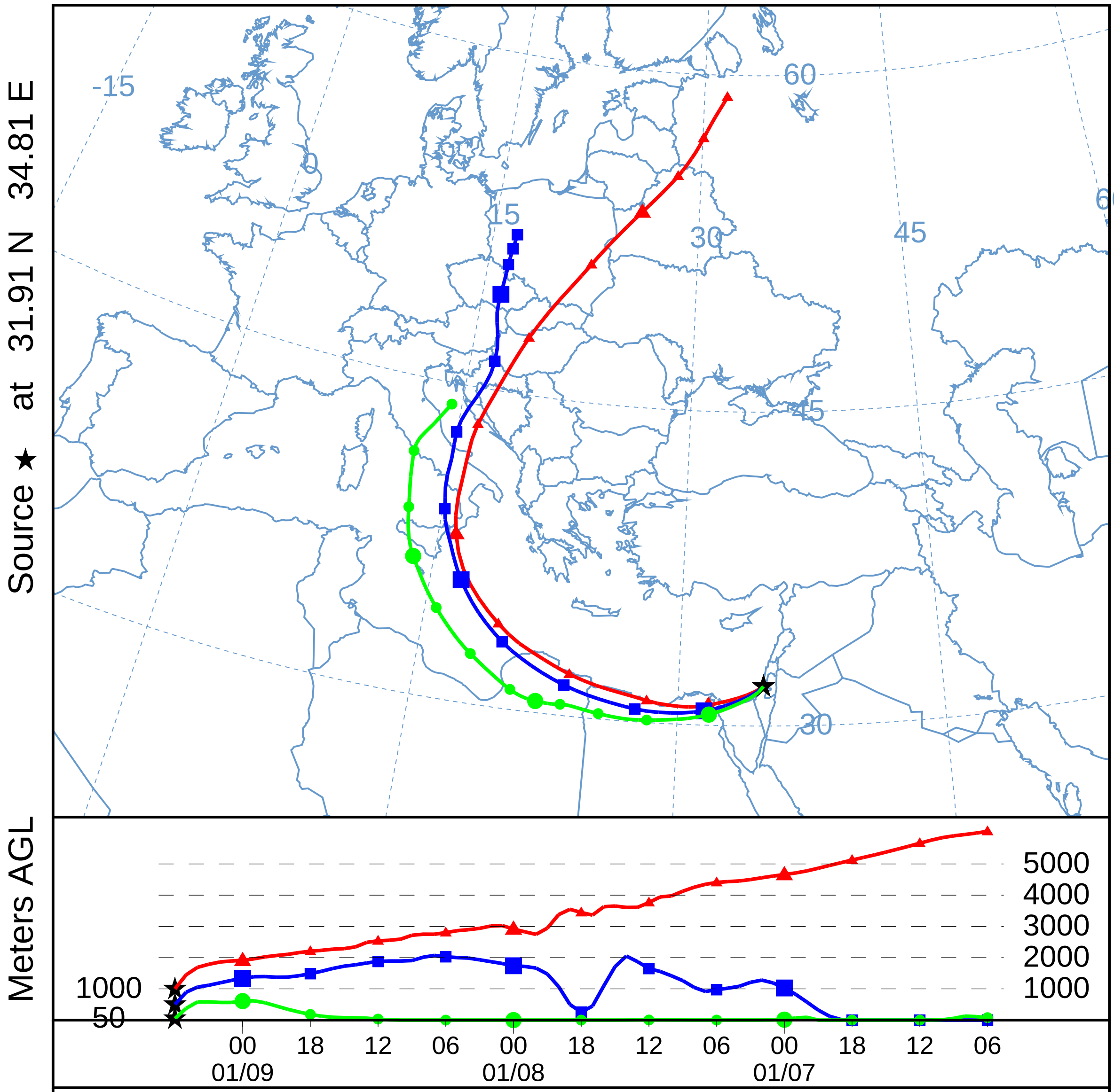

Job ID: 177154 Job Start: Sun Jun 2 12:45:16 UTC 2019
Source 1 lat.: 31.907269 lon.: 34.810664 hgts: 1000, 500, 50 m AGL

Trajectory Direction: Backward Duration: 72 hrs
Vertical Motion Calculation Method: Model Vertical Velocity
Meteorology: 0000Z 8 Jan 2017 - GDAS1
